# Supplementary material for: Genomic and Phenotypic Characterization of CHO 4BGD Cells with Quad Knockout and Overexpression of Two Housekeeping Genes That Allow for Metabolic Selection and Extended Fed-Batch Culturing
Source: Cells. 2025 May 11;14(10):692. doi: 10.3390/cells14100692 (PMC12110749; doi:10.3390/cells14100692)
Supplement: Supplementary file 1 [file cells-14-00692-s001.zip › Supporting_Figure_S1.pdf]

Fig. 1D

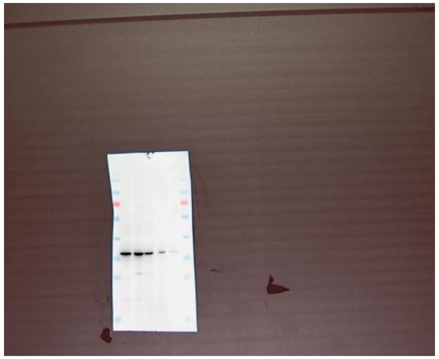

Bak

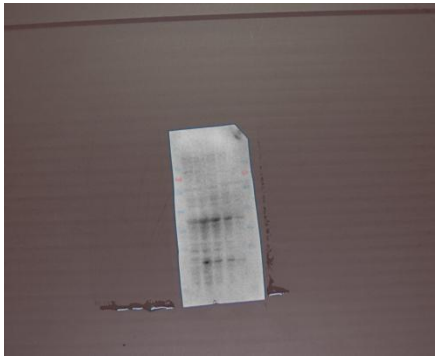

Bax

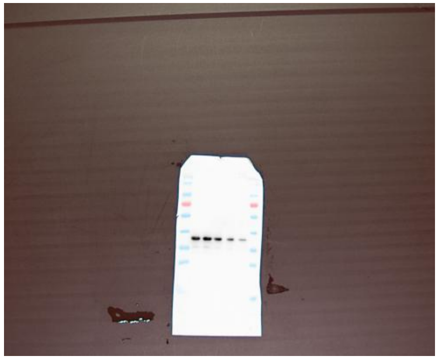

GADPH

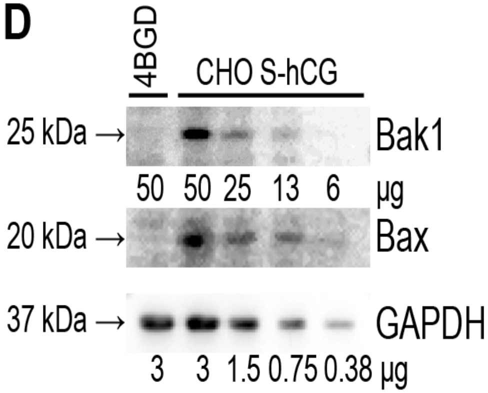

Fig.2D Bcl-2

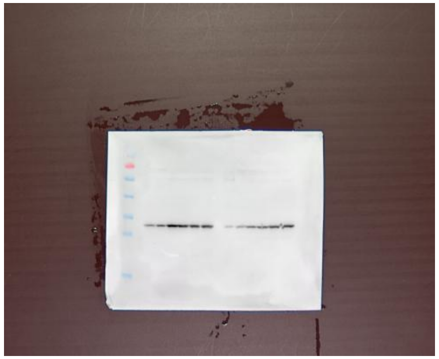

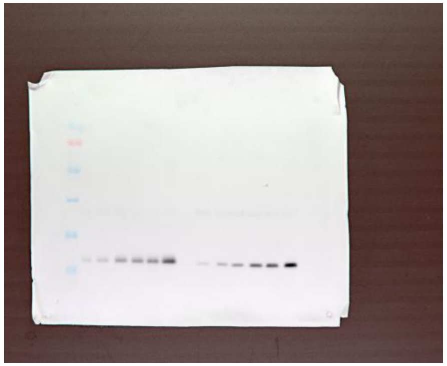

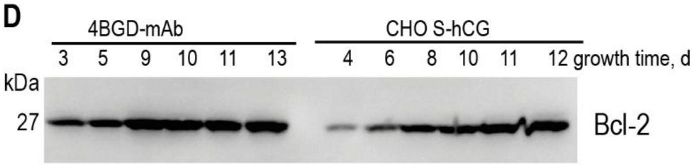

25 mkg/lane

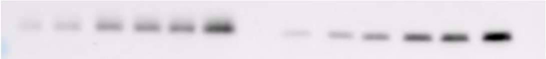

Experiment 1

Experiment 2

In the article 10 mkg/lane

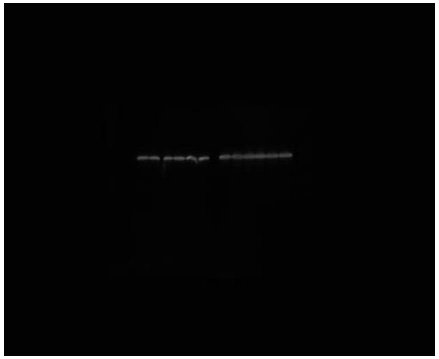

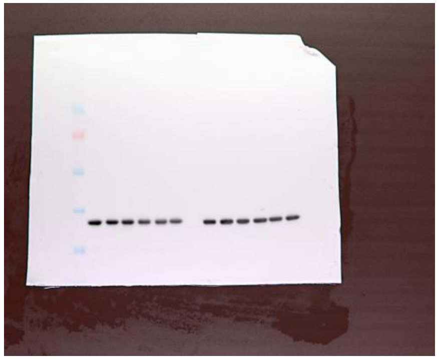

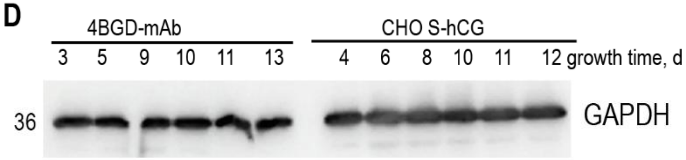

Fig.2D Beclin-1

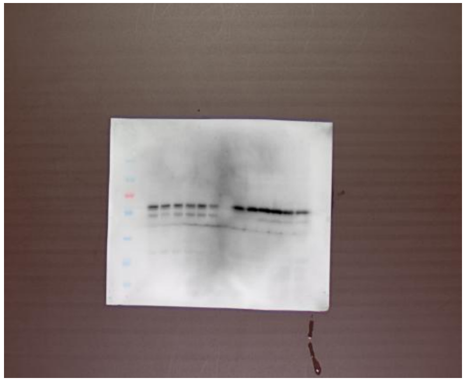

Experiment 1

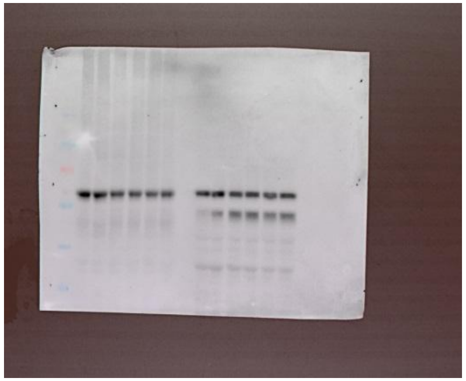

Experiment 2

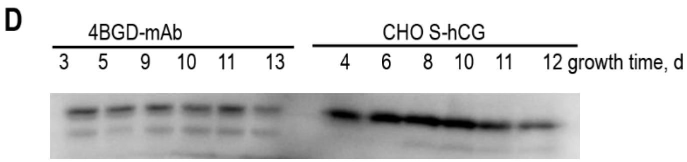

Experiment 1

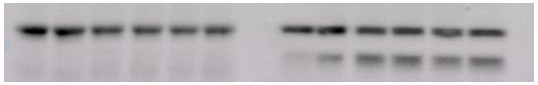

Experiment 2

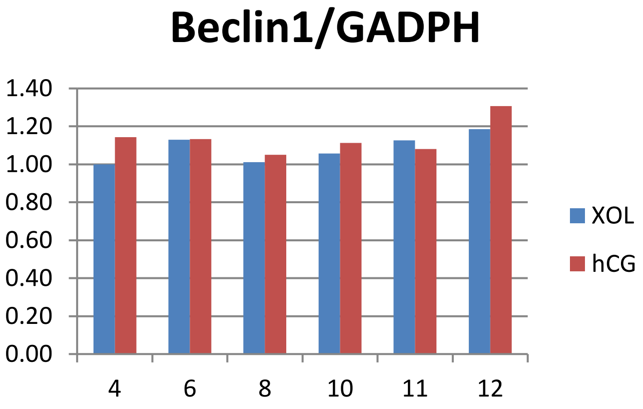

Experiment 2 data

Fig.2D LC3

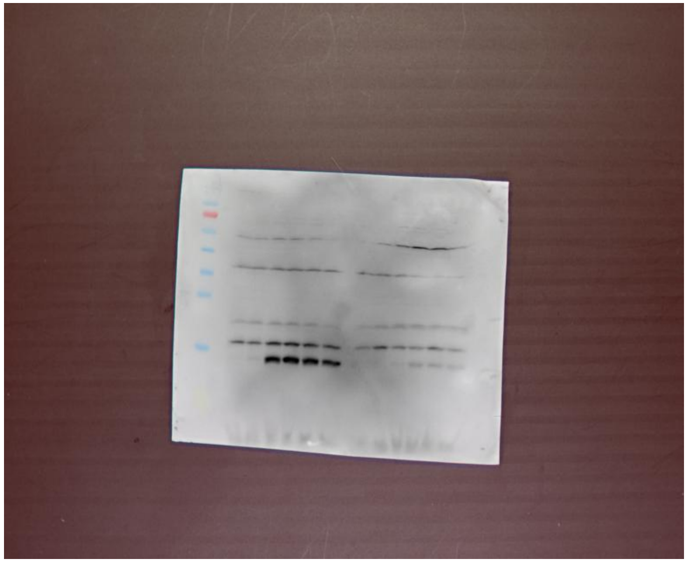

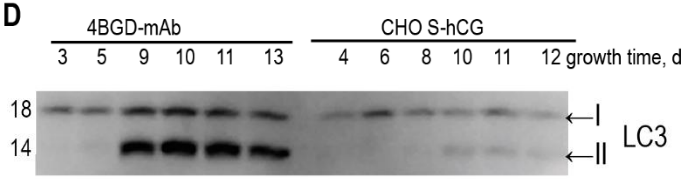

D
4BGD-mAb
CHO S-hCG
3 5 9 10 11 13
4 6 8 10 11 12 growth time, d
18
14
←I
←II
LC3

Fig. 1C

Experiment 1 Experiment 2

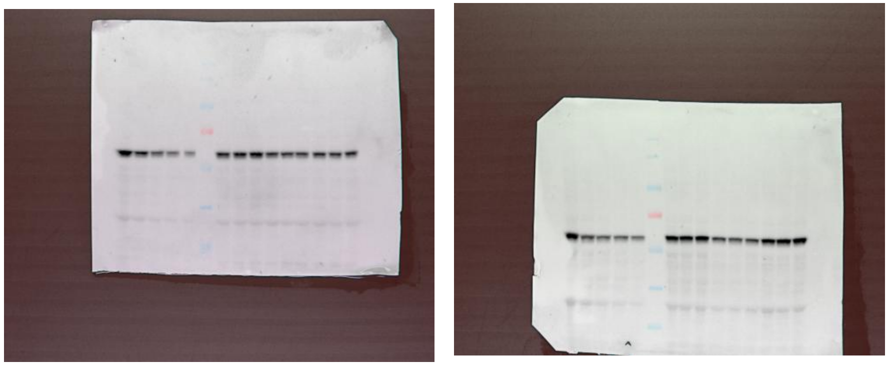

Beclin

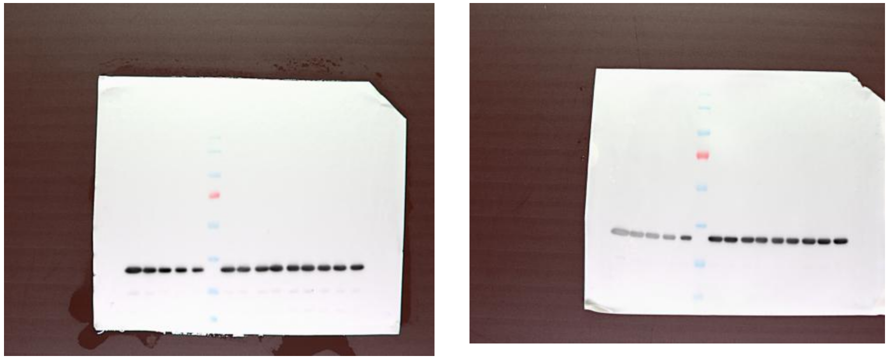

GADPH

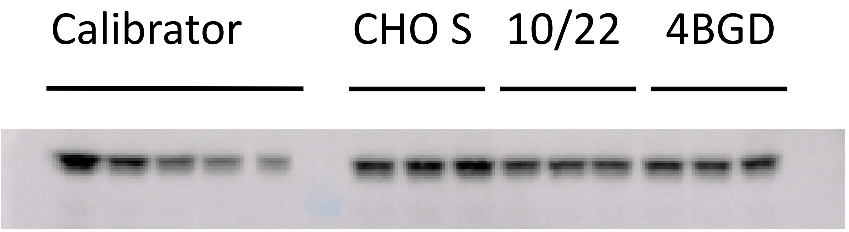

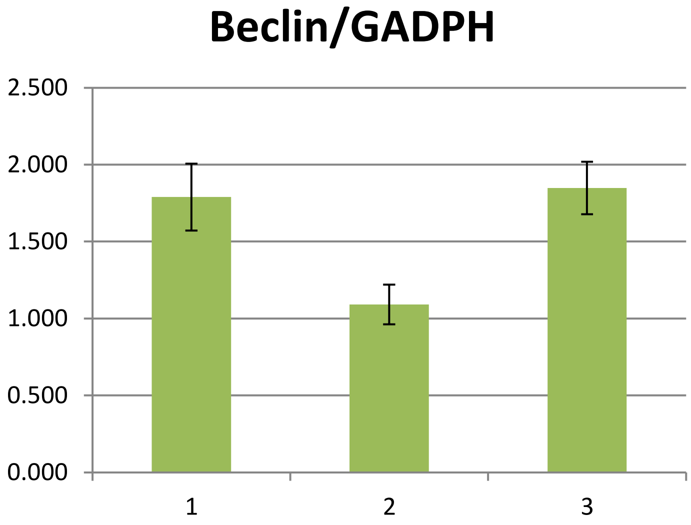

p-values	Beclin 1	GADPH	B/G
S vs 10/22	0.0005	0.0323	0.0089
S vs 4BGD	0.1208	0.2746	0.7304
10/22 vs 4BGD	0.0001	0.0047	0.0036

Fig. 1C

Experiment 1

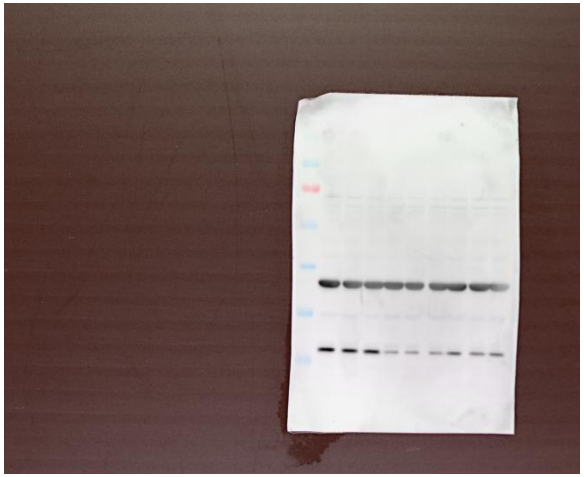

Experiment 2

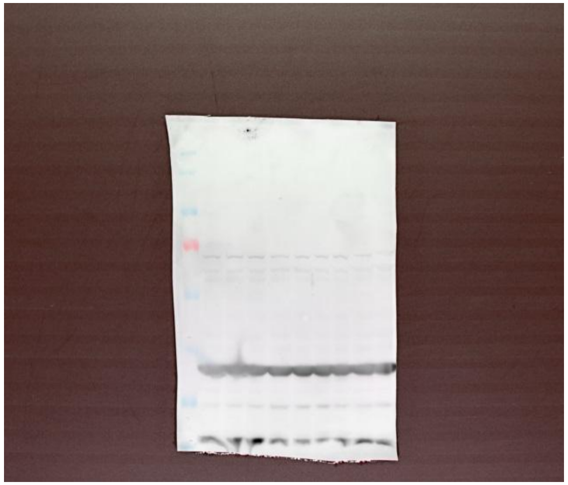

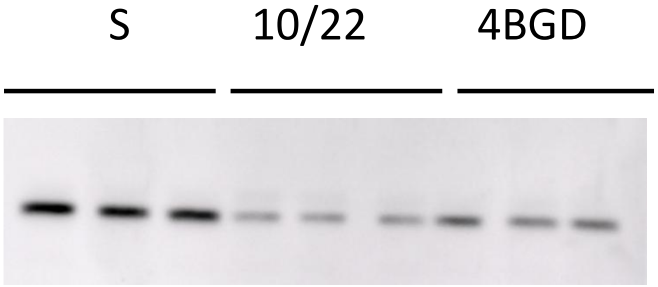

BCL2/GADPH

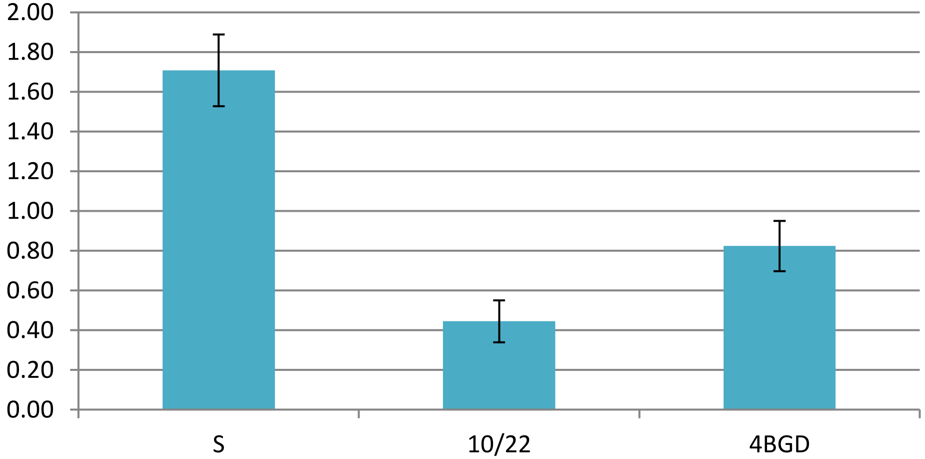

p-values	Bcl2/GAPGH
S vs 10/22	0.0005
S vs 4BGD	0.0022
10/22 vs 4BGD	0.0179
